# Supplementary material for: Multi-Prismatic Hollow Cube CeVO4 with Adjustable Wall Thickness Directed towards Photocatalytic CO2 Reduction to CO
Source: Nanomaterials (Basel). 2023 Jan 10;13(2):283. doi: 10.3390/nano13020283 (PMC9867036; doi:10.3390/nano13020283)
Supplement: Supplementary file 1 [file nanomaterials-13-00283-s001.zip › nanomaterials-2126448-supplementary.pdf]

Multi-prismatic Hollow Cube $CeVO_4$ with Adjustable Thickness Towards Photocatalytic CO_2 Reduction Enhancement

Supplemental information

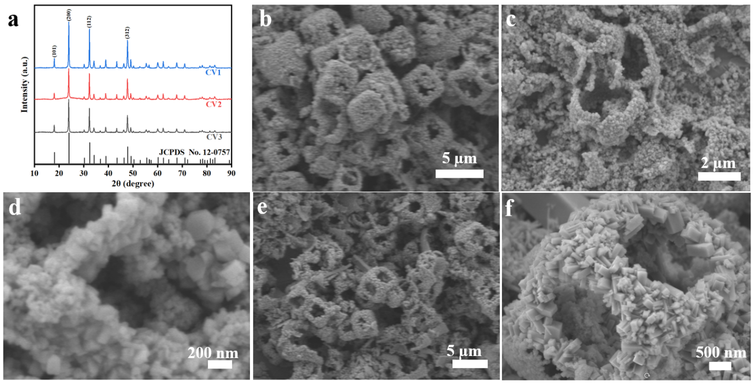

Figure S1. (a) XRD patterns of CV1, CV2, CV3 and tetragonal $CeVO_4$ (JCPDS No. 12-0757); different resolution of SEM images of CV2 (b), CV1 (c, d) and CV3 (e, f).

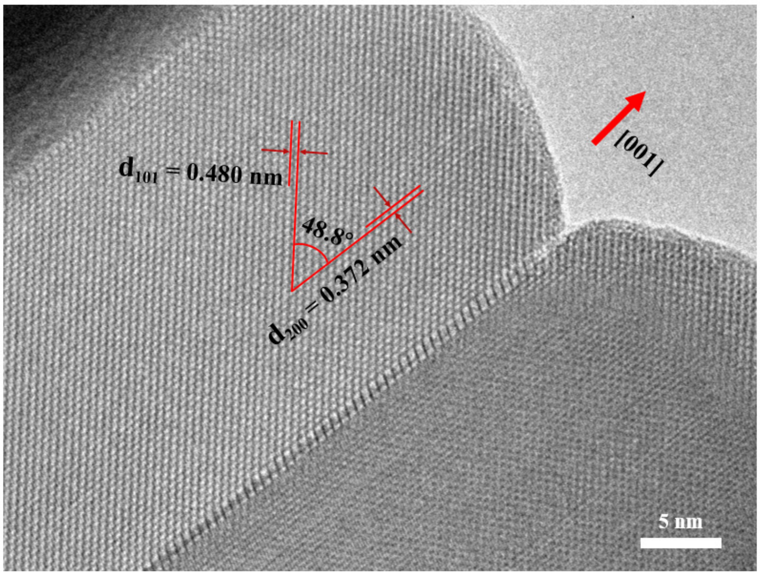

Figure S2. HRTEM image of CV2.

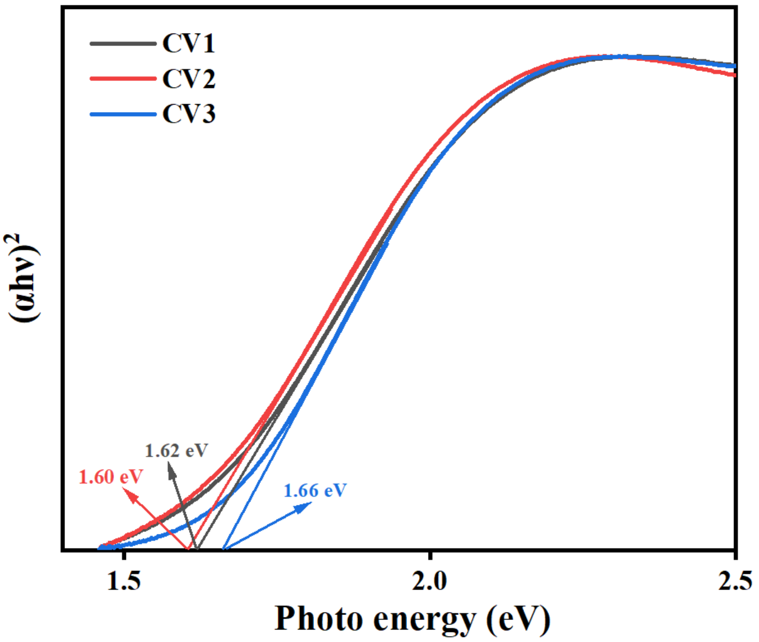

Figure S3. Corresponding plots of transformed Kubelka-Munk function for CV1, CV2 and CV3.
